# Prevalence and significance of early repolarisation in a black African population: data of 246 individuals with cardiovascular morbidity

**DOI:** 10.5830/CVJA-2013-063

**Published:** 2013-10

**Authors:** Aime Bonny, Dominique Noah Noah, Sylvie Ndongo Amougou, Cecile Saka

**Affiliations:** Faculty of Medicine and Phamaceutical Sciences, University Hospital of Douala, Douala, Cameroon; Department of Gastroenterology, Central Hospital of Yaoundé, Yaoundé, Cameroon; Services de réanimation et de cardiologie, Centre Hospitalo-Universitaire de Yaoundé, Yaoundé, Cameroon; Laboratory of Electrocardiography, Hôpital Laquintinie, Douala, Cameroon

**Keywords:** early repolarisation, syncope, ethnicity, black Africans

## Abstract

**Background:**

Early repolarisation (ER) is commonly seen on electrocardiograms (ECG). Recent reports have described the relationship between ER and sudden cardiac death (SCD). The prevalence and significance of ER have not been studied in black Africans.

**Methods:**

We matched clinical and ECG records of subjects over 18 years of age who consulted a cardiac unit in two medical centres of Douala, Cameroon. A questionnaire focusing on past history of syncope or family history of sudden unexplained death (SUD) was filled in by each subject. A 12-lead ECG was recorded by a trained nurse and analysed by two independent physicians.

**Results:**

Of the 752 ECGs recorded, we studied 246 index cases. The mean age of subjects was 45 ± 16 years and 53% were female. Almost 57% had hypertension, 41% had palpitations and 18% reported a history of syncope. ER pattern was found in 20% [slurring in three (3%), notching in 13% and both in three (7%)]. ER subjects were younger than those without (41 ± 16 vs 49 ± 16 years, *p* = 0.0048). Lead localisation was predominantly the laterals for the slurring pattern, whereas the inferior and lateral leads were equally involved for the notching pattern. Negative T waves in the infero-lateral leads were associated with ER (*p* = 0.00025). Among the subjects with syncope, 41% displayed ER and 13% did not have ER (*p* = 0.00014). The notching pattern seemed to be associated with syncope (*p* = 0.00011).

**Conclusion:**

Early repolarisation is frequent in black Africans, especially in the setting of cardiovascular morbidity. Early repolarisation may be associated with a past history of syncope, especially the notched pattern.

## Abstract

Early repolarisation (ER) is an electrocardiographic pattern that consists of early onset as well as an elevation of the transitional QRST–ST junction over 0.1 mV (J-point elevation or JPE) between the depolarisation end and the repolarisation onset of the ventricles.^1^,^2^ This electrophysiological phenomenon is recorded in a surface electrocardiogram (ECG) as J-point elevation, followed by ST-segment elevation and frequently T-wave inversion. The J-point elevation displays two types, including the notching and slurring patterns (Fig. 1).

**Fig. 1.**
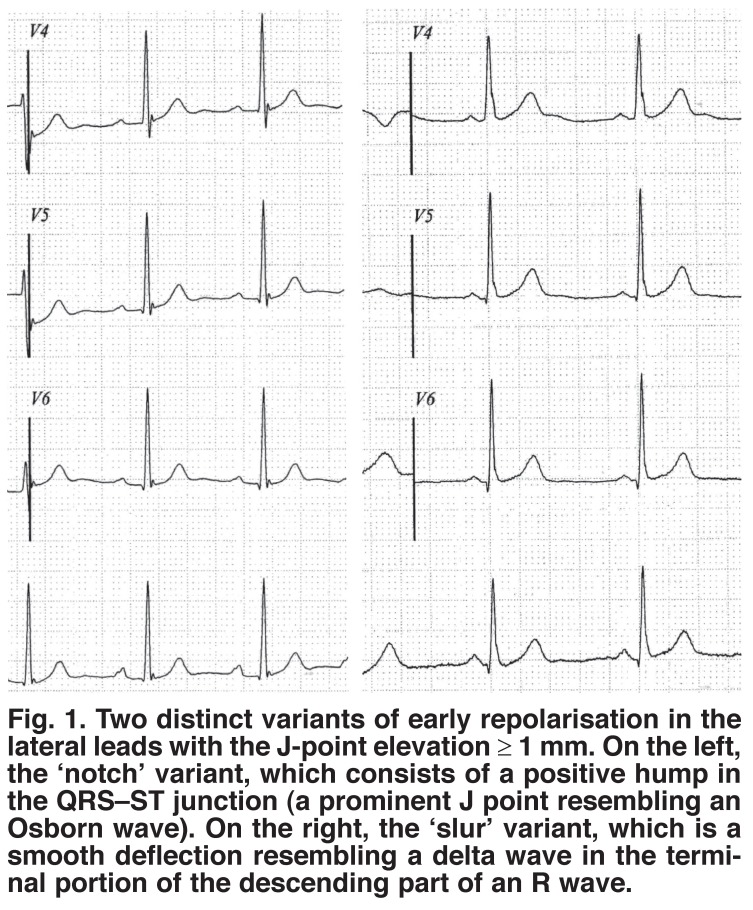
Two distinct variants of early repolarisation in the lateral leads with the J-point elevation ≥ 1 mm. On the left, the ‘notch’ variant, which consists of a positive hump in the QRS–ST junction (a prominent J point resembling an Osborn wave). On the right, the ‘slur’ variant, which is a smooth deflection resembling a delta wave in the terminal portion of the descending part of an R wave.

Interest in this ECG feature is on the increase since it seems not always to be a benign phenotype,^3^-^5^ as was previously thought.^6^-^9^ Indeed, there is growing interest in establishing a correlation between ER and adverse outcomes (Fig. 2).^10^-^13^ As the prevalence of ER is common in the general population and thought to occur more in blacks (Fig. 3),^1^ management of subjects with unexplained syncope and ER in their ECGs may be challenging, particularly in black populations.

**Fig. 2.**
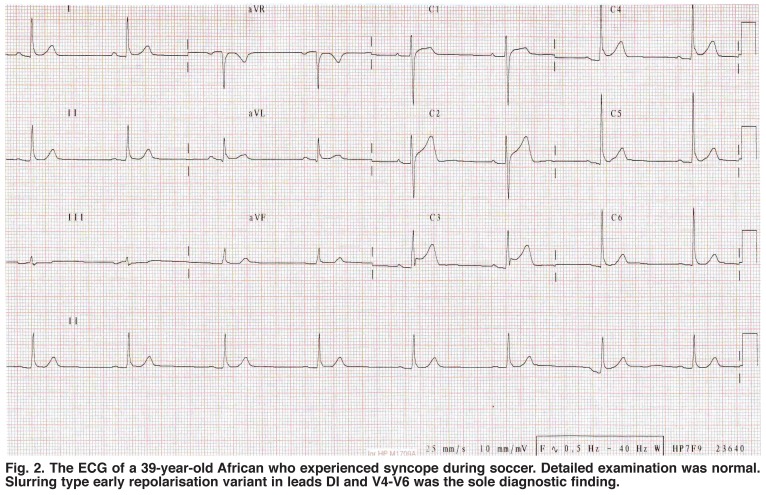
The ECG of a 39-year-old African who experienced syncope during soccer. Detailed examination was normal. Slurring type early repolarisation variant in leads DI and V4‒V6 was the sole diagnostic finding.

**Fig. 3.**
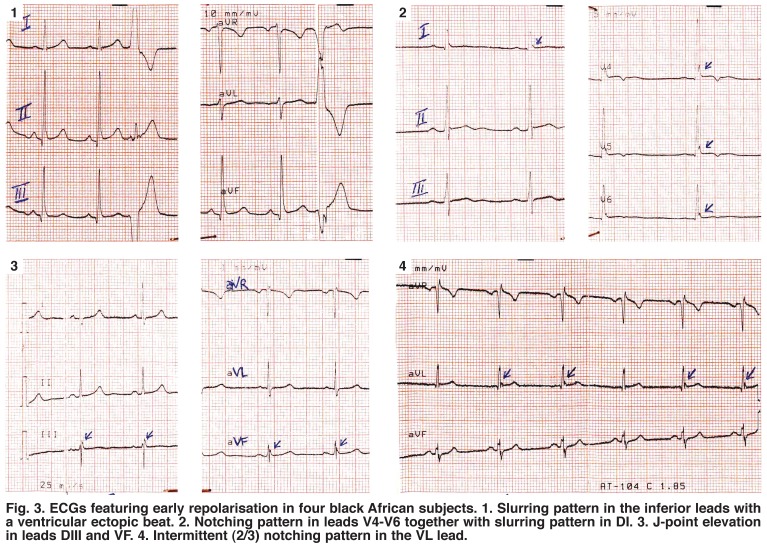
ECGs featuring early repolarisation in four black African subjects. 1. Slurring pattern in the inferior leads with a ventricular ectopic beat. 2. Notching pattern in leads V4‒V6 together with slurring pattern in DI. 3. J-point elevation in leads DIII and VF. 4. Intermittent (2/3) notching pattern in the VL lead.

We aimed to assess the prevalence and significance of ER patterns in a black African population in the setting of tertiary hospitals in Cameroon.

## Methods

Over a two-week period, we matched ECG and clinical data from a sample of patients fulfilling the following inclusion criteria: adult subjects (> 18 years); cardiovascular symptoms or conditions such as palpitations, chest pain, dyspnoea, syncope, hypertension; and ECG done during the study period. The exclusion criteria were: any other causes of transient loss of consciousness such as seizure (Table 1); subjects with low-amplitude ECG waves; subjects with a permanent pacemaker, as ventricular stimulation alters repolarisation; any drug that could potentially modify the QRS, Q-T and ST-T duration and morphology (Table 1); subjects who did not sign the consent form.

**Table 1:** Differential Diagnosis Between Syncope And Seizure

	*Syncope*	*Seizure*
Mechanism	Global transient cerebral hypoperfusion	Abnormal excessive or synchronous neuronal activity
Age (years) at first manifestation	Over 45 if coronary artery disease Mainly < 45 if other cardiac causes such as channelopathies	Less than 45 (often, apart from secondary seizure due to brain damage)
Symptoms		
Before TLOC	Nausea	Aura (funny smell)
	Vomiting	Crying
	Sweating and body cold	
During TLOC	Brief clonic movement (< 15 sec) always secondary to LOC	Prolonged clonic movement at the beginning of LOC Automatism Blue face
After TLOC	Nausea	Prolonged disorientation
	Pale face	Post-event amnesia
	Normal orientation	Weakness, courbature

TLOC = transient loss of consciousness; LOC = loss of consciousness.

Each subject filled in a questionnaire focusing on a past history of transient loss of consciousness (TLOC) or family history of sudden cardiac death (SCD). The diagnosis of syncope was based on the European Society of Cardiology (ESC) guidelines.^10^ Detailed physical evaluation was performed. The ethics committees of both hospitals approved the protocol.

A trained nurse recorded the ECGs using a paper speed of 25 mm/s at 10 mm/mV. After a resting period of at least 5 min, the ECG was registered in the supine position using a numerical electrocardiograph with the capability to review and modify the value of the parameters. Resting 12-lead ECG for each subject was analysed independently by two trained physicians.

A diagnosis of ER was retained if both examiners concluded that at least two consecutive leads displayed a slurring or notching pattern of the descending part of the R wave or a prominent J wave with ST-segment elevation ≥ 1 mm in the lateral or inferior leads. We also paid attention to variation of the J-T segment as ascending, descending or horizontal morphology.

For a practical understanding, we attributed the term type 1 (t1) to the slurring variant of ER and type 2 (t2) to the notching variant. We divided our sample into four groups: ERt1 = early repolarisation type 1, ERt2 = early repolarisation type 2, ERt1t2 = mixed variant, and ER– = the normal pattern of repolarisation.

## Statistical analysis

Continuous variables are expressed as means ± SD and statistical significance was assessed using the unpaired Student’s *t*-test, or Mann-Whitney *U*-test where used to compare mean values between two groups of subjects with ER (ER+) and without (ER–). Categorical variables, were summarised as proportions, and compared using the *χ*^2^ test or Fischer’s exact test. ANOVA and the Kruskal-Wallis test were used to compare mean values between more than two subgroups (ERt1, ERt2, ERt1t2, ER–). The Bonferroni correction was used for adjustment in multiple comparisons. All tests with a two-tailed *p*-value < 0.05 were considered statistically significant. Statistical analysis was performed with SPSS version 11.0.1 software (SPSS Inc)

## Results

Over the two-week period, ECGs were performed in 752 subjects, among whom 248 had cardiac symptoms (palpitations, past history of syncope, chest pain or dyspnoea) or cardiovascular morbidity (hypertension, diabetes, heart failure). Two subjects had low-amplitude ECGs, which did not permit analysis of the repolarisation and they were excluded.

In the remaining 246 subjects studied, the mean age was 45 ± 16 years and 53% were female. The group with ER was younger: 41 ± 16 years versus 49 ± 16 years in subjects without ER (*p* = 0.0048). Baseline characteristics of the sample are shown in Table 2. There were 184 ambulatory subjects (75%).

**Table 2:** Demographic, Clinical And Electrocardiographic Characteristics Of The Sample

	*Aetiology*	*ER+ (n = 49)*	*ER– (n = 197)*	p*-value*
Female (%)	–	53	47	ns
BMI (kg/m^2^)	–	29	28	ns
Hypertension, *n* (%)	–	34 (14)	103 (43)	ns
Diabetes, *n* (%)	–	12 (5)	24 (10)	ns
Palpitation, *n* (%)	–	19 (41)	65 (33)	ns
Syncope, *n* (%)	unknown	19 (41)	26 (13)	0.00014
Acute HF	hypertension	0	2	ns
Family history of SUD, *n* (%)		3 (6.5)	14 (8.6)	ns
T(–) wave, *n* (%)		14 (9)	2 (1)	0.00025
Drug therapy				
Beta-blockers		0	0	
Amiodarone		0	0	
Other anti-arrhythmics		0	0	
Psychotropic drugs		0	0	

ER = early repolarisation; BMI = body mass index; HF = heart failure; SUD = sudden unexpected death; T(–) wave = negative T wave.

Indications for an ECG were hypertension screening in 57% subjects, and palpitations or dizziness in 41%. Two of the in-patients were diagnosed with acute heart failure, and 35 subjects (14.5%) had diabetes. TLOC was reported by 45 (18%) subjects, 19 (41%) in the ER group and 26 (13%) in the group without ER (*p* = 0.00014). A family history of sudden unexpected death (SUD) was reported in three cases (6.5%) in the ER group and in 14 cases (8.5%) in the group with normal repolarisation (*p* = ns). Atrial fibrillation was found in four (1.7%) subjects.

ER was observed in 20% of the population, with the following distribution: slurring pattern in 3.3%, notching pattern in 13%, and both in 3.7%. Among the ER ECGs, we ascertained ascending J-T morphology in eight, descending in five and horizontal in 16 patients.

The slurring pattern was more frequent in the lateral leads (DI = 12%, V6 = 6% and V5 = 4%). The notching pattern was uniformly distributed in the limb and left precordial leads (DI and DII = 20%, VF = 18%, VL = 16%, V5 and V6 = 14%; *p* > 0.05).

The prevalence of negative T waves was 3.7% in this population. In subjects with ER, seven (14%) displayed negative T waves, whereas only two (1%) without ER had this abnormality (*p* = 0.00025).

Subjects with ECGs displaying the ER pattern had a more frequent past history of syncope (*p* = 0.00014). This association was more pronounced in the subgroup of patients with the notching variant of ER pattern (*p* = 0.0001), as shown in Table 3. However, we did not find any correlation between the J-T-segment variations (ascending, descending or horizontal) of ER and syncope. A family history of SUD was not more frequent in subjects with ER features (*p* = ns)

**Table 3:** Comparison Between Subgroups According To Types Of Early Repolarisation

	*ER– (n = 197)*	*ER+ t1 (n = 8)*	*ER+ t2 (n = 32)*	*ER+ t1t2 (n = 9)*	p*-value*
Age (years)	49 ± 16	30 ± 16	45 ± 15	35 ± 14	0.0031
Female, n (%)	107 (54)	4 (50)	14 (44)	3 (33)	ns
Palpitations, n (%)	65 (33)	3 (37)	10 (31)	6 (67)	ns
Syncope, n (%)	26 (13)	3 (37)	15 (47)	1 (11)	0.0001
Family SUD, n (%)	14 (7)	2 (25)	1 (3)	0	ns
T(–) wave, n (%)	2 (1)	1 (< 1)	4 (2)	2 (1)	0.0004

Bonferroni correction was used to calculate the *p*-value. ERV– = absence of early repolarisation; ERV+ = presence of early repolarisation variant; t1 = type 1 or slurring pattern; t2 = type 2 or notching pattern; t1t2 = both patterns; T(–) wave = negative T wave; TLOC = transient loss of consciousness; SUD = sudden unexpected death.

## Discussion

In this study, we observed a high prevalence of ER in patients with a high rate of cardiovascular morbidity. The frequency of ER pattern found was higher than that reported in the general population.^1^,^4^ ER and syncope seemed to be linked, especially the notching pattern. The higher rate of ER in this specific population of subjects at risk for cardiac diseases could indicate a possible risk marker, or an ‘innocent bystander’ of this ECG pattern.

Patients with ER more frequently had negative T waves, suggesting a select population with a poorer prognosis. This finding in such a specific population with a poor prognosis may indicate overlapping of an ER phenotype in cardiovascular outcomes.

This study, as with several others, highlights the question of the relationship between ER and syncope.^11^,^12^ Most studies reporting the clinical significance of ER dealt with the strongest clinical outcome, which is sudden cardiac death due to ventricular tachyarrhythmias. As syncope has a heterogeneity of mechanisms and causes, it is difficult to evaluate its link with ER.

Not all syncopes (e.g. reflex syncope and cardiogenic syncope) have the same explanation in terms of electrophysiological properties of ion channel exchanges driving the formation of J-point elevation (JPE).^13^,^14^ We assumed that non-cardiogenic syncope was not associated with the JPE pattern.

Some studies have described the variation in J-T-segment characteristics after the ER waveforms, the so-called descending, ascending and horizontal morphology, as having prognostic significance.^15^,^16^ Indeed, a horizontal/descending J-T segment in the inferior leads was associated with a significant risk of arrhythmic death, whereas the ascending morphology seemed to have a benign outcome.^16^ In our study, neither ascending nor horizontal/descending patterns were associated with syncope. One potential explanation could be the small sample size. We also emphasise that the heterogeneity of syncope could be a concern.

Arrhythmogenic syncope as a risk marker of SCD merits a prospective investigation in subjects with ER pattern. Our study attempted to put this issue into perspective and discuss the current knowledge on the prevalence and significance of early repolarisation.

## ER and cardiovascular outcomes

ER seems to be associated with a high risk of cardiac arrest.^3^,^17^-^23^ The present study was not designed to confirm this finding.

In the absence of other known causes of syncope, it is difficult to confirm whether ER may be responsible for fainting. Nevertheless, ER has been found in individuals suffering from syncope. 11, 12,24 Indeed, we previously reported the case of exertion syncope with only ER in an apparently healthy young adult.^12^ Maury *et al.*^24^ documented a spontaneous VF by a loop recorder in a patient with an ECG pattern of ER in the inferior leads, who presented with syncope.

The current population was highly specific for being at risk for cardiovascular morbidity, since we studied subjects referred to a cardiac unit for cardiovascular risk factors such as hypertension or diabetes as well as for cardiac-related symptoms (e.g. loss of consciousness). For this reason, the high rate of reported syncope may be explained by the pre-specified clinical behaviour of the sample.

Several studies have reported the high prevalence of ER in young individuals,^21^ with disappearance of this pattern over time (10 years) in 62% of individuals in the Veterans Affairs Palo Alto cohort.^25^ Our findings confirm this result, as the group of subjects with ER was significantly younger than those without ER. We hypothesise that ER pattern, which is an electrocardiographic manifestation of mismatch between outward and inward trans-membrane ionic currents, is a juvenile phenotype, which progressively disappears with time.

It would be of interest to establish the distribution of J-T-segment morphologies (ascending, horizontal/descending) according to age group. This information may add data to aid understanding of risk stratification of ER pattern, as the odds of arrhythmic outcomes follows the life expectancy of each index individual.

## Prognostic markers of ER

Recent studies suggest that ER is not as benign as was earlier believed. Therefore a careful evaluation of carriers, especially in those with syncope or ventricular arrhythmias and/or a family history of sudden cardiac death is justified.^14^ Many studies have attempted to identify markers of malignancy, helping to clarify the clinical significance of this syndrome.

In individuals in whom ER was associated with a high rate of cardiac death, J-point elevation ≥ 0.2 mV was found to be a strong predictor.^4^ In the cohort studied by Tikkanen *et al.*, inferior lead involvement was the sole risk marker of cardiac and sudden arrhythmia deaths, whereas a rapidly ascending ST segment after the J point seems to be a benign variant.^4^,^16^ The lateral leads were also incriminated in other studies.^9^,^17^ Therefore, given the definition of ER syndrome, which excludes the right precordial leads, lead localisations have marginal significance.

Few trials have compared gender and origin of individuals exhibiting ER.^26^ In the ARIC study, whites and females were at higher risk of SCD.^27^

Two distinct patterns of J-point elevation are recognised. Patel *et al*. found the notching (a positive J-point deflection inscribed on the S wave) pattern seemed to be of great value in the risk stratification of individuals with ventricular vulnerability.^28^ Maury *et al*.^29^ found the same significance of the notching pattern, and in his cohort from the city of Toulouse (France), the long-term follow up of a general population has linked the notching variant of ER to cardiac mortality.

Although the notched variant has been shown to have a malignant outcome, the slurred (a smooth transition from QRS to ST segment) variant of repolarisation may confer the same risk as reported by Haruta *et al*.^17^ Our study, although retrospective in design, clearly emphasised a strong association between the notching variant of ER and a history of syncope in index cases of individuals presenting to the tertiary medical centres for cardiac morbidity with a high prevalence of past history of syncope.

## Study limitations

The strength of this study was that although ER is claimed to be more prevalent in black American individuals,^1^ to our knowledge, the prevalence and significance of ER pattern in a black African population has been understudied. However, some limitations merit consideration. First, the study population was a relatively small, select population of black African subjects with cardiovascular morbidity. Second, although the patient interrogation was blinded to the ECG results, the diagnosis of syncope was made retrospectively and based on only the patient’s interrogation. Therefore, it was difficult to distinguish cardiogenic syncope among all causes. Third, this was a cross-sectional rather than a cohort evaluation of ER. Therefore, we could not test the relationship between ER and cardiovascular morbidity and mortality, as was done by several other studies.

## Conclusion

Early repolarisation is a common finding on ECGs of black African individuals presenting for cardiovascular morbidity. The notching rather than the slurring variant was significantly associated with a past history of syncope. J-T-segment morphology was not reported to be linked to syncope. The prognostic significance of ER needs to be established in a prospective fashion.
